# Complete Genome Sequence of the Sourdough Isolate *Lactobacillus zymae* ACA-DC 3411

**DOI:** 10.1128/genomeA.00699-17

**Published:** 2017-07-27

**Authors:** Maria Kazou, Voula Alexandraki, Bruno Pot, Effie Tsakalidou, Konstantinos Papadimitriou

**Affiliations:** a Laboratory of Dairy Research, Department of Food Science and Human Nutrition, Agricultural University of Athens, Athens, Greece; b Research Group of Industrial Microbiology and Food Biotechnology (IMDO), Vrije Universiteit Brussel, Brussels, Belgium

## Abstract

*Lactobacillus zymae* is a Gram-positive lactic acid bacterium belonging to the *Lactobacillus brevis* clade. Here, we report the first complete genome sequence of *L. zymae* ACA-DC 3411, which was isolated from traditional Greek wheat sourdough. Whole-genome analysis may reveal adaptive traits of strain ACA-DC 3411 in the sourdough ecosystem.

## GENOME ANNOUNCEMENT

*Lactobacillus zymae* is a heterofermentative lactic acid bacterium (LAB) species found in fermented foods (1–4), which was transferred from the *Lactobacillus buchneri* clade to the *Lactobacillus brevis* clade, according to a recent 16S rRNA phylogenetic analysis of lactobacilli (5). *L. zymae* ACA-DC 3411 was isolated from traditional Greek wheat sourdough manufactured without baker’s yeast (3, 4). Sourdough has a complex microflora consisting of LAB and yeast species, with lactobacilli being among the most significant group of microorganisms in sourdough fermentation. LAB are mainly involved in dough acidification, whereas yeasts and heterofermentative LAB species participate in the leavening process (6). Analysis of the ACA-DC 3411 genome could prove useful to understand its adaptation in the sourdough environment.

Whole-genome sequencing was performed using the Illumina HiSeq 2000 platform and three paired-end libraries with insert sizes of 500 bp, 2,000 bp, and 6,000 bp at the Beijing Genomics Institute (BGI Co., Ltd., Hong Kong). After filtering, the reads were assembled with the SOAPdenovo version 2.04 software, and the resulting contigs were placed into superscaffolds (7, 8). The assembly was validated using the whole-genome optical map of the strain (9). The map was generated at Microbion SRL (Verona, Italy), and the alignment between the assembly and the optical map was created with the Argus optical mapping system (OpGen Technologies, Inc., Madison, WI). Prediction of protein-coding genes was carried out using Prodigal (10), MetaGeneAnnotator (11) FGENESB (12), and RAST version 2.0, with RAST also being used for the genome annotation and prediction of rRNA and tRNA genes (13). Furthermore, genes were evaluated with the GenePRIMP pipeline for annotation anomalies, including putative pseudogenes (14). Functional annotation of the genome was performed with the WebMGA server (15), the IslandViewer 4 Web-based resource (16), the Phobius Web server (17), and the Pfam database (18) for COG annotation, genomic islands, genes with signal peptides and transmembrane helices, and genes with Pfam domains, respectively.

The genome sequence of ACA-DC 3411 consisted of 2,734,129 bp, with a G+C content of 52.9%. A total of 2,584 genes were identified in the genome, including 2,424 protein-coding genes, 91 potential pseudogenes, 15 rRNA genes, and 54 tRNA genes. According to the COG results, 1,930 protein-coding genes (approximately 80%) were assigned to a putative functional category, with the most abundant being related to replication, recombination, and repair (14%). Moreover, 19 integrated genomic islands were predicted in the ACA-DC 3411 genome, containing a total of 265 genes potentially acquired through horizontal gene transfer. Fifty-six of these genes code for hypothetical proteins, and the rest are of variable function. Additionally, the analysis revealed that the genome contains also 285 protein-coding genes with signal peptides, 545 with transmembrane helices, and 2,012 with Pfam domains. Further analysis of the ACA-DC 3411 genome may reveal the technological potential of the strain for sourdough fermentation.

### Accession number(s).

The genome sequence of *L. zymae* ACA-DC 3411 is deposited at the European Nucleotide Archive under the accession number LT854705.
